# Association of Self-Reported Physical Fitness with Pregnancy Related Symptoms the GESTAFIT Project

**DOI:** 10.3390/ijerph18073345

**Published:** 2021-03-24

**Authors:** Nuria Marín-Jiménez, Milkana Borges-Cosic, Olga Ocón-Hernández, Irene Coll-Risco, Marta Flor-Alemany, Laura Baena-García, José Castro-Piñero, Virginia A. Aparicio

**Affiliations:** 1 Department of Physical Education and Sport, Faculty of Sport Sciences, University of Granada, 18071 Granada, Spain; nuria.marin@uca.es (N.M.-J.); milkanaa@hotmail.com (M.B.-C.); 2 Sport and Health University Research Institute (IMUDS), 18007 Granada, Spain; irecollrisco@gmail.com (I.C.-R.); virginiaparicio@ugr.es (V.A.A.); 3 GALENO Research Group, Department of Physical Education, Faculty of Education Sciences, University of Cádiz, 11519 Cádiz, Spain; jose.castro@uca.es; 4 Biomedical Research and Innovation Institute of Cádiz (INiBICA) Research Unit, 11009 Cádiz, Spain; 5 Biohealth Research Institute in Granada (ibs.GRANADA), 18012 Granada, Spain; ooconh@ugr.es; 6 Gynaecology and Obstetrics Unit, ‘San Cecilio’ University Hospital, 18016 Granada, Spain; 7 Department of Physiology, Faculty of Pharmacy, University of Granada, 18011 Granada, Spain; 8 Institute of Nutrition and Food Technology (INYTA), Biomedical Research Centre (CIBM), University of Granada, 18016 Granada, Spain; 9 Department of Nursing, Faculty of Health Sciences, University of Granada, 18016 Granada, Spain; lbaenagarcia@ugr.es

**Keywords:** International Fitness Scale, gestation, strength, flexibility, cardiorespiratory fitness, agility, pregnancy discomfort.

## Abstract

We explored the association of physical fitness (PF) with pregnancy-related symptoms, at the 16th and 34th gestational weeks (g.w.). The International Fitness Scale and the Pregnancy Symptoms Inventory were employed to assess self-reported PF and pregnancy-related symptoms, respectively. At the 16th g.w. greater self-reported overall PF was associated with lower incidence of urinary frequency (*p* = 0.020); greater overall PF, cardiorespiratory fitness (CRF), muscular strength and speed-agility were associated with lower incidence of tiredness-fatigue (all, *p* < 0.05); greater overall PF and speed-agility were associated with lower incidence of poor sleep (both, *p* < 0.05); greater CRF and flexibility were associated with lower limitations by tiredness-fatigue (both, *p* < 0.05); and greater flexibility was associated with lower limitations by poor sleep (*p* = 0.021). At the 34th g.w. greater self-reported overall PF, CRF and muscular strength were associated with lower incidence of tiredness-fatigue (all, *p* < 0.05); greater CRF was associated with lower incidence of poor sleep (*p* = 0.019); and, greater flexibility was associated with lower incidence of increased vaginal discharge (*p* = 0.023). Adequate levels of PF, especially CRF, may help women to cope with the most endorsed pregnancy-related symptoms and its limitations, especially tiredness-fatigue and poor sleep.

## 1. Introduction

Pregnancy is a physiological stage characterized for physical and psychological changes that, in the vast majority of women, involve symptoms related to the pregnancy course [1,2]. In fact, more than thirty physical and psychological symptoms have been described in normal pregnancies and the most commonly reported symptoms are being fatigued, increased urinary frequency, pain (such as breast pain, headache or back pain), vaginal discharge or sleep disruption-insomnia [3,4,5,6]. The duration and severity of these pregnancy-related symptoms vary significantly among women, which may occur throughout the whole pregnancy course although most notably during either first (early pregnancy) and third trimester (late pregnancy) [5,7]. These symptoms may cause discomfort and negatively affect the pregnant women quality of life [1,8] by limiting their activities of daily living [5]. Therefore, the early detection of pregnancy-related symptoms and their limitations in activities of daily living is essential to design alternative therapies aimed at minimize these negative effects on the pregnancy course progress [1]. 

Pharmacological treatment may minimize pregnancy-related symptomatology, although is not a desirable and save option due to the potential side effects for the fetus [9]. Consequently, pregnant women are more likely to use non-pharmacological methods to manage pregnancy-related physical symptoms [5]. In this sense, physical fitness (PF), and its components, is a powerful health indicator in the general population [10,11,12,13] and the screening of PF in the clinical practice could provide valuable information related to maternal and fetal health during pregnancy, as previously reported [14,15,16,17]. Indeed, PF is described as "*the ability to carry out daily tasks with vigor and alertness, without undue fatigue and with ample energy to enjoy leisure-time pursuits and to meet unforeseen emergencies*" [13], highlighting *“without undue fatigue”* as an important element especially during pregnancy, when fatigue is known as a common symptom. Moreover, health-related PF is composed of cardiorespiratory function, muscle function (such as muscular strength), motor abilities (such as agility and speed) and indices of morphology (such as joint flexibility) [13,18]. And the level between each of them can vary from person to person, so all or some of these components could be related to lower pregnancy-related symptoms, and their impact on limitations on activities of daily living [13].

When an objective assessment of PF is not available due to time-cost limitations [19], self-reported PF has been proposed as a feasible and valid alternative in different populations [20,21,22], including pregnant women [14,17]. Therefore, the International Fitness Scale (IFIS) [20] covers the perception of pregnant women on these components of physical fitness in a few minutes.

Greater self-reported PF levels during pregnancy have been previously associated with better quality of life [14] and lower pregnancy-related pain [17]. As a result, screening of self-reported PF during pregnancy could be a useful, quick and inexpensive tool to initiate an effective non-pharmacological treatment, like adapted physical exercise programs [23].

Therefore, we hypothesized that self-reported PF could be also associated with other pregnancy-related symptoms that may exert a negative impact on the pregnancy course, such as increased fatigue, sleep disturbances or urinary frequency. As far as we are aware, whether greater PF levels during pregnancy may be associated with lower pregnancy-related symptoms, and their impact on limitations on activities of daily living has not been reported. Consequently, the aim of the present study was to analyze the association of self-reported PF level with pregnancy-related symptoms, and its limitations on activities of daily living along pregnancy. 

## 2. Materials and Methods

### 2.1. Study Design and Participants

The present cross-sectional study is part of the GESTAFIT project randomized controlled trial, where a novel supervised exercise intervention program was developed. The complete procedures, the inclusion-exclusion criteria and the sample size calculation of the project have been published elsewhere [24]. A total of 159 Spanish pregnant women (32.9 ± 4.6 years old) enrolled in this study in three waves (from November 2015 to March 2017), for feasibility reasons. The participants were recruited by the research team between the 11–13th gestational week (g.w.), during their first gynecologist checkup at the “San Cecilio” University Hospital (Granada, Spain). A written informed consent was signed by all interested participants after being informed about the study aims and procedures. 

This study was approved by the Ethics Committee on Clinical Research of Granada, Regional Government of Andalusia, Spain (code: GESFIT-0448-N-15). 

### 2.2. Procedures

After the recruitment, participants were invited to participate in the study at the Sport and Health University Research Institute (Granada, Spain). Assessments were carried out at the 16th and 34th g.w. (±2 weeks). Firstly, participants filled an auto-administered anamnesis assessing their sociodemographic characteristics and medical and reproductive history. Subsequently, the participants filled questionnaires assessing their self-reported PF and the Pregnancy Symptoms Inventory (PSI), among others. 

### 2.3. Sociodemographic and Clinical Data

Sociodemographic data including age, marital status, educational level, working status and number of children were collected. 

### 2.4. Anthropometry and Body Composition

Body weight and height were assessed using a scale (InBody R20; Biospace, Seoul, Korea) and a stadiometer (Seca 22, Hamburg, Germany), respectively. Body mass index (BMI) was calculated as weight (kg) divided by squared height (m^2^). 

### 2.5. Physical Fitness

The IFIS [20] was employed to assess self-reported PF level. The IFIS is composed of five Likert-scale questions asking the participants about their perceived overall physical fitness, CRF, muscular strength, speed-agility and flexibility in comparison with their non-pregnant counterparts. The participants rated their physical fitness levels as “very poor = 1”, “poor = 2”, “average = 3”, “good = 4” and “very good = 5” [20]. The IFIS has been previously validated [14] and used in pregnant population [17]. 

### 2.6. Pregnancy Related-Symptoms

The PSI [3], in the Spanish validated version [4], was used to assess the nature and the frequency of the effects of pregnancy-related symptoms The PSI is a 41-items Likert inventory, self-administered questionnaire that assesses the pregnancy-related symptoms and how frequent these symptoms limit the activities of daily living of pregnant women. Firstly, participants responded to each symptom as “never”, “rarely”, “sometimes” or “often” occurred. A symptom was considered endorsed if the participant indicated “sometimes” or “often”. Consequently, they completed the second part of the questionnaire evaluating how affected they were by that symptom, as “not limited at all”, “limit a little” or “limit a lot” their activities of daily living. A symptom was considered as a limitation if the participant indicated “limit a little” or “limit a lot”. Previous studies summarized the prevalence of these symptoms and its limitations as a “top three” [5] or “top four-five” [3,4] most reported. Therefore, we chose to explore the "top four" most commonly reported pregnancy-related symptoms [3,4].

### 2.7. Statistical Analyses

All analyses were performed using the Statistical Package for Social Sciences (IBM SPSS Statistics for Windows, version 22.0, Armonk, NY, USA) and the level of significance was set at *p* ≤ 0.05. Descriptive statistics [mean (standard deviation) for quantitative variables and number of women (%) for categorical variables] were used to describe baseline characteristics of the participants. Frequencies of each symptom and limitation were determined by the number of those women experiencing a particular symptom/limitation. Linear regression analyses were performed to explore the association of self-reported overall PF, CRF, muscular strength, speed-agility and flexibility with the “top four” frequently reported pregnancy-related symptoms and limitations in each evaluation (at the 16th and 34th g.w.). We controlled for potential confounding such as maternal age and BMI. The models were additionally adjusted for the exercise intervention program (control or intervention group) in those variables assesssed at the 34th g.w., in order to correct the possible effect of the exercise program performed in the GESTAFIT project [24]. We additionally adjusted the models for number of children, and results remained the same (data not shown).

## 3. Results

**Figure 1:**
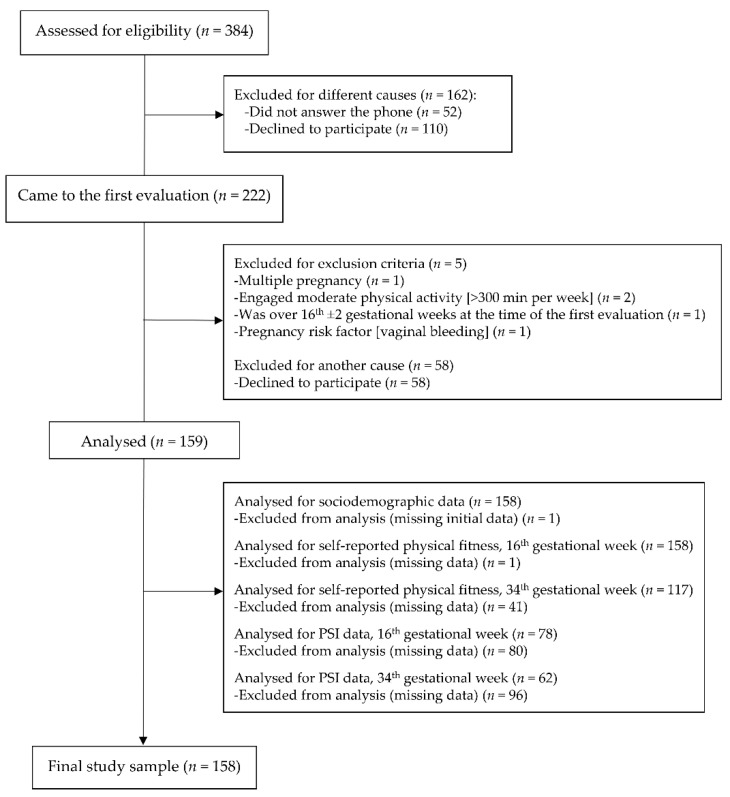
Flow diagram of study participants.

The final sample size was composed of 159 Spanish pregnant women. Nonetheless, the PSI questionnaire was included in the second wave of recruitment, since we considered that information meaningful after the pregnancy-symptoms experienced by our pregnant women and reported to our research team. Therefore, the PSI sample was *n* = 78, at the 16th g.w., and *n* = 62, at the 34th g.w. Moreover, some of them did not attend the second evaluation (at the 34th g.w.) or did not return all the questionnaires duly completed, which meant a loss of data in some other outcomes (see Figure 1).

Sociodemographic and clinical characteristics of the participants are shown in Table 1. The “top four” pregnancy symptoms, reported sometimes or often, at the 16th g.w. were: urinary frequency (92.3%), tiredness-fatigue (85.9%), poor sleep (74.4%) and breast pain (70.5%). At the 34th g.w.: poor sleep (91.9%), urinary frequency (90.3%), tiredness-fatigue (87.1%) and increased vaginal discharge (72.6%). The “top four” frequency of their limitations in activities of daily living at 16th g.w. were: tiredness-fatigue (68.0%), poor sleep (63.2%), urinary frequency (54.6%) and headache (52.7%). At the 34th g.w.: poor sleep (83.6%), tiredness-fatigue (80.7%), urinary frequency (64.5%) and back pain (58.9%).

The total 41-items self-reported pregnancy symptoms reported sometimes or often, and the frequency of their limitations in activities of daily living at the 16th and 34th g.w. are shown in Table S1. 

The partial correlations of self-reported overall PF and its components with pregnancy symptoms and limitations to activities of daily living at the 16th and 34th g.w. are shown in Table S2.

The linear regression model assessing the association of self-reported overall PF and its components with the “top four” reported pregnancy-related symptoms and limitations at the 16th and 34th g.w. are shown in Table 2. At the 16th g.w., greater self-reported overall PF was associated with lower incidence of urinary frequency (β = −0.30, *p* = 0.020). Greater self-reported overall PF, CRF, muscular strength and speed-agility were associated with lower incidence of tiredness-fatigue (β = −0.31, *p* = 0.018; β = −0.29, *p* = 0.018; β = −0.25, *p* = 0.031 and β = −0.34, *p* = 0.006, respectively). Greater self-reported overall PF and speed-agility were associated with lower incidence of poor sleep (β = −0.46, *p* < 0.001 and β = −0.31, *p* =0.014, respectively). Greater self-reported CRF and flexibility were associated with lower limitations by tiredness-fatigue (β = −0.34, *p* =0.006 and β=−0.25, *p* = 0.035, respectively). Finally, greater self-reported flexibility was associated with lower limitations by poor sleep (β = −0.28, *p* = 0.021). 

At the 34th g.w., greater self-reported overall PF, CRF and muscular strength were associated with lower incidence of tiredness-fatigue (β = −0.32, *p* = 0.013; β = −0.33, *p* = 0.012 and β = −0.29, *p* = 0.032, respectively). Greater self-reported CRF was associated with lower incidence of poor sleep (β = −0.31, *p* = 0.019). Finally, greater self-reported flexibility was associated with lower incidence of increased vaginal discharge (β = −0.31, *p* = 0.023).

## 4. Discussion

Our findings indicate that greater overall self-reported PF was associated with less incidence of pregnancy-related symptoms and its limitations in activities of daily living. Specifically, greater self-reported overall PF, CRF, muscular strength and speed-agility were associated with lower incidence of tiredness-fatigue. Moreover, greater self-reported overall PF, CRF and speed-agility were associated with lower incidence of poor sleep. Attending its limitations on activities of daily living, greater self-reported CRF and flexibility were associated with less tiredness-fatigue during the early second trimester of pregnancy. 

The mean age of the women at the time of the recruitment was 32.9 ± 4.6 years old (in the average of Spaniards pregnancy age [25]). Pregnant women showed average level of overall self-reported PF and all its components throughout the pregnancy course. Top prevalence of pregnancy-related symptoms reported by our sample of pregnant women were similar to those found in the original PSI version [3], the Spanish-validated version [4], and other studies investigating pregnancy-related symptoms [5,7]. In our study, tiredness-fatigue and poor sleep were specially reported as endorsed pregnancy-related symptoms and limitations in activities of daily living along the pregnancy course. The findings of the present study shown that greater PF levels were associated with lower incidence of these two commonly reported pregnancy-related symptoms. 

Fatigue has been associated with pregnancy complications and fears, such as depression, risk of caesarean section, fear of childbirth and weak maternal-infant attachment, which may seriously impact materno-fetal health and quality of life [8]. Moreover, physical, anatomical, physiological and hormonal changes associated with pregnancy, fetal movements and the size of the uterus, may negatively affect sleep patterns [5,7]. In addition, a high percentage of pregnant women experience fatigue in all trimesters [5], aggravated also by sleep disruptions [5]. 

Other symptoms, such as increased urinary frequency and vaginal discharge are common urogenital system complaints throughout pregnancy [5], also confirmed in our study sample. The integumentary and vascular systems involve altered levels of circulating hormones, increased intravascular volume, and compression from the enlarging uterus underlie the complex physiological adaptations to the pregnancy course [26], which may also contribute to poor sleep and increased fatigue feelings. Insomuch as these pregnancy-related changes/symptoms are interconnected, it is plausible that improving some of them may also exert a positive impact on others. In this sense, our results show that pregnant women with greater self-reported overall PF levels and its components may experience both, lower incidence and lower limitations in activities of daily living due to tiredness-fatigue and poor sleep. 

Since our study is the first to analyze the relationship of PF levels with pregnancy-related symptoms, it is not possible to compare the present findings with other similar studies. Nevertheless, pregnancy-related symptoms that caused the largest effect on women’s lives such as fatigue-tiredness, sleep disruptions-insomnia, and increased urination need to be deeply explored and understood, preventing women from taking unnecessary pharmacological treatment. Some possible hypothesis about the mechanisms involved in these associations could be proposed. Regarding fatigue-tiredness, there is a lack of studies exploring the influence of PF on fatigue during pregnancy. In general population, greater physical activity levels have been associated with about 40% reduced risk in experiencing low energy and fatigue [27]. In pregnant women, a recent systematic review and meta-analysis concluded that following a supervised exercise program during pregnancy reduces fatigue [8]. Moreover, a previous study, conducted in general population, showed that those participants with lower self-reported PF had poorer sleep quality [28]. Specifically, poor sleep quality was associated with lower levels of muscular strength, CRF and flexibility [29]. Our results showed that those pregnant women with greater self-reported overall PF (especially CRF and speed-agility) reported lower incidence of poor sleep. Moreover, those with greater self-reported flexibility also reported lower limitations due to poor sleep. Although further research is needed to elucidate the mechanisms, a possible explanation promoting reduced fatigue and better sleep quality is that exercise plays a role in brain circuitry, neurotransmitters and neuromodulators, regulating motor functioning and mediating mood disturbances, through monoamines, histamine or gabapentin-mediated neurotransmission [27]. In addition, exercise plays a role in the thermoregulatory mechanism, improving vasodilation, decreasing cortisol levels or exerting well-being and a mental-calm state [28], consequently decreasing sleep disturbances. 

Regarding urogenital problems, one possible explanation is that pregnant women may have a weak pelvic floor, as it has been studied that a higher BMI is associated with weaker pelvic floor muscles [30], and our participants had an average BMI of 25 kg/ m^2^ at the 16th g.w. Moreover, lower PF levels may also exert a negative effect, especially in the later stages of pregnancy, due to the pressure of the growing fetus on the muscles of the utero muscles [31]. In fact, other factors, such as low PF levels and sleep disturbances are also significant contributors to the development of fatigue [32].

Greater PF levels are improved by practicing physical activity or exercise [23,33]. The specialized 2019-Canadian Guidelines for physical exercise during pregnancy indicate that “there may be periods when following the guidelines is not possible due to fatigue and/or discomforts of pregnancy” [23]. In fact, common barriers to be physically active during pregnancy include discomforts of pregnancy, among others [34]. Nevertheless, our results suggest that pregnant women with greater self-reported PF levels may suffer from lower frequency and severity of these pregnancy-related symptoms, such as fatigue and discomfort. Therefore, we encourage pregnant women to reach greater PF levels in order to deal with these symptoms and limitations through pregnancy course, although some modification to exercise routines may be necessary [33]. In this sense, women should accomplish with at least 150 min of moderate intensity physical activity per week, combining aerobic, resistance-strength activities plus pelvic floor training, to obtain meaningful health benefits and reductions in pregnancy-related complications [23]. This should be especially mandatory during pregnancy since we have previously found that only 22% of the Spanish pregnant women complied with these recommendations [35].

Some limitations of the present study should be acknowledged. Firstly, the cross-sectional design precludes determination of causality. For instance, it is possible that women with greater tiredness-fatigue are less likely to be physically active or vice-versa [34] (although changes in physical fitness levels need time to increase/decrease, not fluctuating acutely). Moreover, our study sample was relatively small, mainly due to the design of the PSI, which allows to answer only if the symptom is considered as frequent [3,4], and the fact that we did not included this questionnaire in our first recruitment wave [24]. Regarding strengths, most studies until date have only assessed a few pregnancy-related symptoms, while we have measured a wide range of them (41 pregnancy-related symptoms and limitations).

## 5. Conclusions

Greater self-reported overall PF and its different components, especially CRF, have shown a strong relationship with lower incidence and limitations of the most common pregnancy-related symptoms, particularly tiredness-fatigue and poor sleep. Pregnant women may benefit from exercise programs focused on improving PF in order to experience a less unpleasant pregnancy due to these symptoms. Moreover, health care providers, physical therapists and physical exercise specialists should screen PF levels and encourage pregnant women to reach adequate PF levels during this stage. 

## Figures and Tables

**Table 1:** Sociodemographic and clinical characteristics of the participants.

Maternal Characteristics	n	Mean ± SD
Age (years)	158	32.9 ± 4.6
Height (cm)	157	163 ± 6.21
Weight at the 16th gestational week (kg)	157	67.0 ± 11.8
Weight at the 34th gestational week (kg)	123	74.6 ± 10.8
Body max index at the 16th gestational week (kg/m^2^)	157	24.9 ± 4.14
Marital status	158	n (%)
Married		91 (57.6)
Single		66 (41.8)
Divorced/separated/widow		1 (0.6)
Educational level	158	
Primary or high-school		37 (23.4)
Specialized training		27 (17.1)
University degree		94 (59.5)
Working status	158	
Homework/unemployed		48 (30.4)
Partial-time employed/student		41 (25.9)
Full-time employed		69 (43.7)
Parity	158	n (%)
Nulliparous		96 (60.8)
Multiparous		62 (39.2)
Previous abortions		66 (42.0)
Self-reported physical fitness * (1–5)		
16th gestational week	158	
Overall physical fitness		3.2±0.7
Cardiorespiratory fitness		2.5±0.9
Muscular strength		3.1±0.7
Speed-agility		3.0±0.8
Flexibility		3.1±1.0
34th gestational week	117	
Overall physical fitness		3.3±0.7
Cardiorespiratory fitness		2.6±0.8
Muscular strength		3.2±0.7
Speed-agility		2.9±0.7
Flexibility		3.1±1.0
Top four pregnancy symptoms (0–3)	78	n (%)
16th gestational week		
Urinary frequency		72 (92.3)
Tiredness-fatigue		67 (85.9)
Poor sleep		58 (74.4)
Breast pain		55 (70.5)
34th gestational week	62	
Poor sleep		57 (91.9)
Urinary frequency		56 (90.3)
Tiredness-fatigue		54 (87.1)
Increased vaginal discharge		45 (72.6)
Top four limitations ** (0–2)		
16th gestational week		
Tiredness-fatigue	78	53 (68.0)
Poor sleep	76	48 (63.2)
Urinary frequency	77	42 (54.6)
Headache	72	38 (52.7)
34th gestational week		
Poor sleep	61	51 (83.6)
Tiredness-fatigue	62	50 (80.7)
Urinary frequency	62	40 (64.5)
Back pain	56	33 (58.9)

SD, Standard Deviation. * Self-reported physical fitness varies from 1 (“very poor”) to 5 (“very good”). ** As the number of women responding questions varied, the denominator is displayed for each symptom (n). Women who did not experience any of the symptoms did not answer questions about their limitations.

**Table 2:** Linear regression coefficients assessing the association of self-reported physical fitness and frequent symptoms and limitations at the 16th and 34th gestational weeks.

Items	Pregnancy Symptoms								Limitations							
	16th Gestational Week				34th Gestational Week				16th Gestational Week				34th Gestational Week			
	β	B	SE	*p*	β	B	SE	p *	β	B	SE	*p*	β	B	SE	p *
	Overall physical fitness															
Urinary frequency	−0.30	−0.22	0.09	0.020 *	−0.09	−0.08	0.11	0.486	−0.18	−0.15	0.11	0.177	−0.07	−0.07	0.13	0.586
Tiredness-fatigue	−0.31	−0.28	0.12	0.018 *	−0.33	−0.32	0.13	0.013 *	−0.16	−0.14	0.12	0.246	−0.26	−0.26	0.13	0.052
Poor sleep	−0.46	−0.53	0.14	<0.001*	−0.26	−0.26	0.13	0.052	−0.14	−0.12	0.12	0.300	−0.05	−0.05	0.14	0.705
Breast pain	−0.05	−0.06	0.17	0.709	*NA*	*NA*	*NA*	*NA*	*NA*	*NA*	*NA*	*NA*	*NA*	*NA*	*NA*	*NA*
Increased vaginal discharge	*NA*	*NA*	*NA*	*NA*	−0.21	−0.26	0.17	0.134	*NA*	*NA*	*NA*	*NA*	*NA*	*NA*	*NA*	*NA*
Headache	*NA*	*NA*	*NA*	*NA*	*NA*	*NA*	*NA*	*NA*	−0.14	−0.13	0.14	0.324	*NA*	*NA*	*NA*	*NA*
Back pain	*NA*	*NA*	*NA*	*NA*	*NA*	*NA*	*NA*	*NA*	*NA*	*NA*	*NA*	*NA*	−0.05	−0.05	0.14	0.742
	Cardiorespiratory fitness															
Urinary frequency	−0.13	−0.08	0.07	0.278	−0.04	−0.04	0.10	0.728	−0.24	−0.17	0.09	0.059	0.10	0.10	0.12	0.436
Tiredness-fatigue	−0.29	−0.22	0.09	0.018 *	−0.33	−0.30	0.11	0.012 *	−0.34	−0.26	0.09	0.006 *	−0.21	−0.19	0.12	0.120
Poor sleep	−0.20	−0.19	0.12	0.108	−0.31	−0.28	0.12	0.019 *	−0.04	−0.03	0.09	0.769	−0.04	−0.03	0.12	0.779
Breast pain	−0.07	−0.07	0.13	0.593	*NA*	*NA*	*NA*	*NA*	*NA*	*NA*	*NA*	*NA*	*NA*	*NA*	*NA*	*NA*
Increased vaginal discharge	*NA*	*NA*	*NA*	*NA*	−0.24	−0.28	0.15	0.076	*NA*	*NA*	*NA*	*NA*	*NA*	*NA*	*NA*	*NA*
Headache	*NA*	*NA*	*NA*	*NA*	*NA*	*NA*	*NA*	*NA*	−0.15	−0.12	0.11	0.252	*NA*	*NA*	*NA*	*NA*
Back pain	*NA*	*NA*	*NA*	*NA*	*NA*	*NA*	*NA*	*NA*	*NA*	*NA*	*NA*	*NA*	−0.18	−0.18	0.13	0.182
	Muscular strength															
Urinary frequency	−0.09	−0.06	0.08	0.44	0.06	0.06	0.11	0.611	−0.10	−0.08	0.10	0.415	−0.07	−0.07	0.13	0.586
Tiredness-fatigue	−0.25	−0.22	0.10	0.031 *	−0.29	−0.27	0.12	0.032 *	−0.20	−0.17	0.10	0.094	−0.22	−0.21	0.13	0.102
Poor sleep	−0.20	−0.22	0.13	0.089	−0.07	−0.07	0.13	0.584	0.01	0.01	0.10	0.939	−0.20	−0.18	0.13	0.153
Breast pain	0.03	0.04	0.14	0.802	*NA*	*NA*	*NA*	*NA*	*NA*	*NA*	*NA*	*NA*	*NA*	*NA*	*NA*	*NA*
Increased vaginal discharge	*NA*	*NA*	*NA*	*NA*	0.05	0.06	0.17	0.720	*NA*	*NA*	*NA*	*NA*	*NA*	*NA*	*NA*	*NA*
Headache	*NA*	*NA*	*NA*	*NA*	*NA*	*NA*	*NA*	*NA*	−0.10	−0.09	0.12	0.450	*NA*	*NA*	*NA*	*NA*
Back pain	*NA*	*NA*	*NA*	*NA*	*NA*	*NA*	*NA*	*NA*	*NA*	*NA*	*NA*	*NA*	−0.14	−0.14	0.14	0.328
	Speed-agility															
Urinary frequency	0.16	0.11	0.08	0.189	0.10	0.09	0.10	0.407	0.22	0.17	0.10	0.085	0.03	0.03	0.12	0.801
Tiredness-fatigue	−0.34	−0.28	0.10	0.006 *	−0.18	−0.16	0.12	0.193	−0.18	−0.15	0.11	0.163	−0.15	−0.14	0.12	0.255
Poor sleep	−0.31	−0.32	0.13	0.014 *	−0.06	−0.06	0.12	0.643	−0.15	−0.12	0.10	0.254	−0.05	−0.04	0.12	0.727
Breast pain	0.18	0.21	0.15	0.114	*NA*	*NA*	*NA*	*NA*	*NA*	*NA*	*NA*	*NA*	*NA*	*NA*	*NA*	*NA*
Increased vaginal discharge	*NA*	*NA*	*NA*	*NA*	−0.15	−0.17	0.15	0.290	*NA*	*NA*	*NA*	*NA*	*NA*	*NA*	*NA*	*NA*
Headache	*NA*	*NA*	*NA*	*NA*	*NA*	*NA*	*NA*	*NA*	−0.09	−0.09	0.13	0.481	*NA*	*NA*	*NA*	*NA*
Back pain	*NA*	*NA*	*NA*	*NA*	*NA*	*NA*	*NA*	*NA*	*NA*	*NA*	*NA*	*NA*	0.05	0.04	0.13	0.727
	Flexibility															
Urinary frequency	−0.09	−0.05	0.07	0.456	−0.16	−0.12	0.09	0.203	−0.06	−0.04	0.08	0.619	−0.16	−0.13	0.11	0.223
Tiredness-fatigue	−0.15	−0.10	0.08	0.213	−0.10	−0.08	0.10	0.450	−0.25	−0.18	0.08	0.035 *	−0.04	−0.03	0.11	0.766
Poor sleep	−0.02	−0.02	0.11	0.854	−0.20	−0.16	0.11	0.143	−0.28	−0.19	0.08	0.021 *	−0.15	−0.12	0.10	0.267
Breast pain	−0.14	−0.14	0.12	0.260	*NA*	*NA*	*NA*	*NA*	*NA*	*NA*	*NA*	*NA*	*NA*	*NA*	*NA*	*NA*
Increased vaginal discharge	*NA*	*NA*	*NA*	*NA*	−0.31	−0.31	0.13	0.023 *	*NA*	*NA*	*NA*	*NA*	*NA*	*NA*	*NA*	*NA*
Headache	*NA*	*NA*	*NA*	*NA*	*NA*	*NA*	*NA*	*NA*	−0.08	−0.07	0.10	.517	*NA*	*NA*	*NA*	*NA*
Back pain	*NA*	*NA*	*NA*	*NA*	*NA*	*NA*	*NA*	*NA*	*NA*	*NA*	*NA*	*NA*	−0.13	−0.10	0.11	0.358

* (Significant values, *p* < 0.05). β, standardized regression coefficient; B, non-standardized regression coefficient; SE, Standard Error. Model adjusted for maternal age, body max index at the 16th or 34th gestational weeks and exercise intervention at the 34th gestational week. NA, not applicable.

## Data Availability

Data presented in this study will be made available from the corresponding author under reasonable request.

## Supplementary Materials

The following are available online at https://www.mdpi.com/1660-4601/18/7/3345/s1, Table S1: Prevalence of self-reported pregnancy symptoms and limitations to activities of daily living at 16th (*n* = 78) and 34th (*n* = 62) gestational weeks. Table S2: Partial correlations of self-reported overall PF and its components with pregnancy symptoms and limitations to activities of daily living at the 16th and 34th gestational weeks.
