# Supplementary material for: Association of Self-Reported Physical Fitness with Pregnancy Related Symptoms the GESTAFIT Project
Source: Int J Environ Res Public Health. 2021 Mar 24;18(7):3345. doi: 10.3390/ijerph18073345 (PMC8036338; doi:10.3390/ijerph18073345)
Supplement: Supplementary file 1 [file ijerph-18-03345-s001.pdf]

Supplementary Table S1. Prevalence of self-reported pregnancy symptoms and limitations to activities of daily living at the 16th (n = 78) and 34th (n = 62) gestational weeks.

Items	Pregnancy symptoms						Limitations*							
	16th gestational week			34th gestational week			16th gestational week				34th gestational week			
	Sometimes	Often	Total prevalence	Sometimes	Often	Total prevalence	n	Limit a little	Limit a lot	Total prevalence	n	Limit a little	Limit a lot	Total prevalence
**Tiredness-fatigue**	38.5%	47.4%	85.9%	33.9%	53.2%	87.1%	78	44.9%	23.1%	68.0%	62	48.4%	32.3%	80.7%
**Nausea**	25.6%	28.2%	53.8%	16.1%	1.6%	17.7%	70	30.0%	17.1%	47.1%	45	13.3%	0.0%	13.3%
**Vomiting**	11.5%	12.8%	24.3%	3.2%	1.6%	4.8%	55	18.2%	12.7%	30.9%	45	6.7%	2.2%	8.9%
**Reflux**	17.9%	15.4%	33.3%	30.6%	33.9%	64.5%	65	12.3%	6.2%	18.5%	53	32.1%	9.4%	41.5%
**Constipation**	29.5%	25.6%	55.1%	21.0%	11.3%	32.3%	67	19.4%	7.5%	26.9%	53	20.8%	0.0%	20.8%
**Hemorrhoids**	6.4%	5.1%	11.5%	9.7%	6.5%	16.2%	47	8.5%	0.0%	8.5%	45	8.9%	0.0%	8.9%
**Dry mouth**	19.2%	16.7%	35.9%	35.5%	4.8%	40.3%	58	8.6%	3.4%	12.0%	54	11.1%	0.0%	11.1%
**Food cravings**	41.0%	5.1%	46.1%	29.0%	1.6%	30.6%	69	1.9%	0.0%	1.9%	56	3.6%	0.0%	3.6%
**Poor sleep**	44.9%	29.5%	74.4%	25.8%	66.1%	91.9%	76	47.4%	15.8%	63.2%	61	44.3%	39.3%	83.6%
**Restless legs**	15.4%	9.0%	24.4%	19.4%	21.0%	40.4%	52	9.6%	3.8%	13.4%	50	26.0%	12.0%	38.0%
**Leg cramps**	10.3%	0.0%	10.3%	37.1%	14.5%	51.6%	46	8.7%	2.2%	10.9%	56	32.1%	7.1%	39.2%
**Snoring**	17.9%	2.6%	20.5%	9.7%	6.5%	16.2%	51	3.9%	0.0%	3.9%	51	7.8%	0.0%	7.8%
**Urinary frequency**	25.6%	66.7%	92.3%	22.6%	67.7%	90.3%	77	42.9%	11.7%	54.6%	62	41.9%	22.6%	64.5%
**Incontinence/ leaking urine**	12.8%	3.8%	16.6%	17.7%	12.9%	30.6%	53	5.7%	11.3%	17.0%	48	27.1%	6.3%	33.4%
**Increased vaginal discharge**	37.2%	19.2%	56.4%	37.1%	35.5%	72.6%	70	11.4%	1.4%	12.8%	61	29.5%	1.6%	31.1%
**Thrush**	5.1%	3.8%	8.9%	1.6%	6.5%	8.1%	41	9.8%	7.3%	17.1%	40	7.5%	2.5%	10.0%
**Changes in libido**	32.1%	23.1%	55.2%	45.2%	21.0%	66.2%	69	27.5%	8.7%	36.2%	52	26.9%	13.5%	40.4%
**Painful veins in vagina**	0.0%	2.6%	2.6%	6.5%	4.8%	11.3%	35	5.7%	0.0%	5.7%	40	7.5%	5.0%	12.5%
**Carpel tunnel (numb hands)**	7.7%	5.1%	12.8%	12.9%	12.9%	25.8%	46	8.7%	4.3%	13.0%	45	24.4%	4.4%	28.8%

**Sciatica/ pain down the back of your legs**	12.8%	12.8%	25.6%	30.6%	16.1%	46.7%	51	33.3%	13.7%	47.0%	52	30.8%	19.2%	50.0%
**Back pain**	38.5%	24.4%	62.9%	38.7%	25.8%	64.5%	71	35.2%	11.3%	46.5%	56	33.9%	25.0%	58.9%
**Hip or pelvic pain**	23.1%	6.4%	29.5%	29.0%	22.6%	51.6%	59	20.3%	5.1%	25.4%	53	32.1%	22.6%	54.7%
**Breast pain**	29.5%	41.0%	70.5%	16.1%	12.9%	29.0%	71	25.4%	4.2%	29.6%	48	16.7%	0.0%	16.7%
**Headache**	29.5%	29.5%	59.0%	19.4%	4.8%	24.2%	72	31.9%	20.8%	52.7%	49	24.5%	6.1%	30.6%
**Sore nipples**	25.6%	21.8%	47.4%	16.1%	11.3%	27.4%	59	20.3%	3.4%	23.7%	46	8.7%	0.0%	8.7%
**Dizziness**	17.9%	6.4%	24.3%	19.4%	6.5%	25.9%	57	19.3%	7.0%	26.3%	50	18.0%	10.0%	28.0%
**Fainting**	1.3%	0.0%	1.3%	0.0%	0.0%	0.0%	31	6.5%	0.0%	6.5%	39	0.0%	2.6%	2.6%
**Heart palpitations**	24.4%	5.1%	29.5%	22.6%	4.8%	27.4%	51	11.8%	2.0%	13.8%	46	17.4%	0.0%	17.4%
**Shortness of breath**	12.8%	5.1%	17.9%	29.0%	11.3%	40.3%	50	16.0%	4.0%	20.0%	50	38.0%	10.0%	48.0%
**Taste/smell changes**	26.9%	39.7%	66.6%	11.3%	8.1%	19.4%	73	19.2%	9.6%	28.8%	45	8.9%	0.0%	8.9%
**Forgetfulness**	19.2%	9.0%	28.2%	40.3%	16.1%	56.4%	57	17.5%	5.3%	22.8%	54	20.4%	5.6%	26.0%
**Feeling depressed**	25.6%	2.6%	28.2%	27.4%	1.6%	29.0%	57	15.8%	1.8%	17.6%	49	20.4%	2.0%	22.4%
**Anxiety**	11.5%	7.7%	19.2%	16.1%	4.8%	20.9%	53	15.1%	0.0%	15.1%	48	22.9%	0.0%	22.9%
**Vivid dreams**	23.1%	11.5%	34.6%	35.5%	8.1%	43.6%	65	7.7%	0.0%	7.7%	52	11.5%	1.9%	13.4%
**Altered body image**	26.9%	9.0%	35.9%	35.5%	8.1%	43.6%	58	10.3%	0.0%	10.3%	52	11.5%	1.9%	13.4%
**Greasy skin/acne**	23.1%	16.7%	39.8%	25.8%	22.6%	48.4%	61	9.8%	1.6%	11.4%	49	24.5%	4.1%	28.6%
**Varicose veins**	3.8%	11.5%	15.3%	11.3%	3.2%	14.5%	44	9.1%	6.8%	15.9%	41	4.9%	2.4%	7.3%
**Brownish marks on face**	5.1%	6.4%	11.5%	22.6%	14.5%	37.1%	0	0.0%	0.0%	0.0%	47	21.3%	4.3%	25.6%
**Itchy skin**	26.9%	28.2%	55.1%	11.3%	17.7%	29.0%	67	14.9%	6.0%	20.9%	49	14.3%	0.0%	14.3%
**Changes in nipples**	30.8%	25.6%	56.4%	25.8%	27.4%	53.2%	66	10.6%	1.5%	12.1%	54	16.7%	5.6%	22.3%
**Stretch marks**	9.0%	2.6%	11.6%	30.6%	27.4%	58.0%	44	6.8%	0.0%	6.8%	53	15.1%	0.0%	15.1%

**Swollen hands or feet**	6.4%	3.8%	10.2%	9.7%	6.5%	16.2%	47	14.9%	2.1%	17.0%	41	4.9%	2.4%	7.3%

*As the number of women responding questions varied, the denominator is displayed for each symptom (n). Women who did not experience any of the symptoms did not answer questions about their limitations.

Supplementary Table S2. Partial correlations of self-reported overall PF and its components with pregnancy symptoms and limitations to activities of daily living at the 16th and 34th gestational weeks.

Items	Pregnancy symptoms										Limitations*									
	16th gestational week (n = 74)					34th gestational week (n = 23)					16th gestational week (n = 59)					34th gestational week (n = 31)				
	OPF	CRF	MS	S-A	FLEX	OPF	CRF	MS	S-A	FLEX	OPF	CRF	MS	S-A	FLEX	OPF	CRF	MS	S-A	FLEX
Tiredness-fatigue	**-,277***	**-,279***	**-,255***	-,322	-,149	-,349	-,342	**-,307***	-,193	-,121	-,074	-,293	,113	-,129	-,211	-,188	-,094	-,148	-,273	-,273
Nausea	-,177	-,182	**-,247***	-,163	-,124	-,478	-,314	-,204	**-,297***	-,152	,024	,066	,177	,230	,215	-,197	-,003	**-,369***	-,267	-,018
Vomiting	-,012	-,114	-,057	-,087	,101	-,274	-,104	-,218	-,189	-,009	,033	,060	,168	,062	,074	**-,390***	,035	-,228	-,342	,074
Reflux	,011	,038	,032	-,064	,051	-,248	-,164	-,055	-,062	**-,376****	,094	,007	,419	,124	-,009	-,201	,147	-,165	,008	-,256
Constipation	-,040	-,181	-,071	,096	-,079	-,194	-,077	,137	,051	,130	-,393	-,616	-,307	-,339	-,343	,054	,000	,257	,184	,102
Haemorrhoids	-,131	-,040	**-,315****	,022	,087	-,071	-,314	-,143	,012	,103						-,073	-,159	,020	,075	,068
Dry mouth	-,142	-,141	-,030	-,042	,054	-,292	-,236	-,114	-,161	,183	-,185	-,577	-,182	,005	-,203	**-,461***	-,344	**-,443***	-,490	-,220
Food cravings	,094	,065	,006	-,163	,038	,130	,077	**,321***	,187	,037						-,026	-,096	,028	,074	,064
Poor sleep	**-,408****	-,191	-,202	-,289	-,022	-,253	-,309	-,072	-,062	-,195	-,244	-,214	-,033	-,341	-,318	-,035	-,010	-,147	-,104	**-,382***
Restless legs	-,173	-,124	,026	,036	-,073	-,074	-,130	,012	-,120	-,114	**-,489***	-,172	-,125	-,131	-,024	-,020	-,112	,011	,131	-,166
Leg cramps	,034	-,119	,042	,128	-,055	,105	,183	,175	,140	,032						-,248	,154	-,150	-,276	-,166
Snoring	-,056	-,067	-,032	,029	,141	,082	-,067	,045	-,096	,007	-,005	-,125	-,206	,094	,056	-,040	,051	-,249	-,073	,075
Urinary frequency	**-,273***	-,130	-,093	,157	-,089	-,152	,017	,006	,068	-,206	-,413	-,600	-,350	-,417	-,353	-,110	,067	-,215	-,146	**-,459***

**Incontinence/ leaking urine**	-,023	-,129	,073	-,187	-,126	-,173	-,093	-,001	,025	-,041	-,075	,005	,286	-,386	-,385	-,110	,053	-,082	-,125	**-,378***
**Increased vaginal discharge**	-,180	-,035	-,167	-,239	-,211	-,223	-,248	,021	-,159	**-,316***	-,104	-,041	,346	-,283	-,322	**-,518****	-,173	-,453*	-,475	-,345
**Thrush**	,006	-,094	-,060	,176	,085	-,054	,130	,052	-,093	-,238	-,114	-,391	-,273	,079	,078	-,193	-,006	-,080	-,318	**-,472****
**Changes in libido**	**-,268***	-,201	-,205	-,088	-,017	-,153	,007	-,156	-,149	-,145	-,149	-,021	-,306	-,039	-,037	,055	,086	-,214	,039	-,057
**Painful veins in vagina**	-,009	,055	-,018	-,257	-,235	-,009	,166	,072	-,164	-,134						,065	**,407***	,247	-,053	-,285
**Carpel tunnel (numb hands)**	**-,251***	-,133	**-,333****	-,119	,032	-,212	-,189	-,103	-,144	,001	-,131	,082	-,347	-,088	,141	-,153	-,193	-,356	-,132	-,048
**Sciatica/ pain down the back of your legs**	-,058	**-,267***	-,218	-,283	,018	-,136	-,030	-,136	-,162	-,098	,152	-,312	,166	-,078	-,180	-,129	-,162	-,111	-,297	-,336
**Back pain**	-,021	-,143	,052	-,031	,011	-,250	-,215	-,164	-,108	-,177	,121	-,355	,374	,203	,034	,017	-,239	-,053	,061	-,291
**Hip or pelvic pain**	-,187	-,200	-,064	-,243	,043	-,385	-,261	-,219	-,154	-,118	,005	-,192	,020	-,318	-,369	-,214	-,168	-,063	-,046	**-,463***
**Breast pain**	-,045	-,064	,030	,172	-,134	-,061	-,069	-,018	-,117	-,094	-,011	-,406	-,203	,266	,266	-,350	-,005	-,302	-,379	-,283
**Headache**	-,087	-,046	-,103	,115	,083	-,303	-,313	-,128	-,229	-,153	-,055	-,086	-,003	,151	,217	-,338	-,329	-,104	-,272	**-,403***
**Sore nipples**	-,114	-,166	**-,248***	,102	-,135	-,117	-,168	-,100	,017	,090	-,054	-,450	,006	-,054	-,132	-,026	-,096	,028	,074	,064
**Dizziness**	-,176	-,140	-,022	,005	,081	-,229	-,143	,051	-,093	,045	-,269	-,122	-,208	,135	,284	-,080	-,158	-,094	-,202	-,301
**Fainting**	-,150	-,092	**-,265***	-,013	,076	,028	-,018	-,071	-,006	-,207	-,304	-,137	-,519	,091	,203	,173	-,116	,180	,011	-,185

**Heart palpitations**	-,111	-,050	-,127	-,151	,001	-,205	-,012	-,020	-,205	-,099	-,304	-,266	-,130	-,024	-,124	,033	,207	-,104	,070	-,006
**Shortness of breath**	-,088	-,131	-,138	-,178	-,225	-,291	-,250	-,158	-,105	,102	-,121	-,197	-,268	,188	,103	-,068	-,110	-,256	-,093	-,231
**Taste/smell changes**	-,110	-,128	-,125	,059	,080	-,185	-,304	-,079	-,095	-,044	-,238	-,213	-,046	-,221	-,261	-,006	,002	-,008	,029	,012
**Forgetfulness**	-,138	,097	-,002	-,070	-,010	-,149	-,094	,010	-,039	,054	-,287	-,062	-,488	-,208	-,286	,111	,356	-,114	-,075	-,012
**Feeling depressed**	-,177	-,039	**-,233***	-,028	,088	-,189	-,250	-,243	-,197	-,018	-,338	-,177	-,240	,030	,076	,122	,103	-,114	-,108	,087
**Anxiety**	-,137	-,059	-,138	-,020	-,091	-,348	-,219	-,237	**-,399****	-,133	-,294	-,253	,025	-,050	-,164	-,343	-,123	**-,422***	-,319	-,207
**Vivid dreams**	**,246***	,081	**,467****	-,035	-,028	-,056	,011	-,076	-,241	-,073	-,032	-,102	,087	-,327	-,347	-,348	-,091	-,311	-,455	**-,370***
**Altered body image**	,148	,052	,192	,297	-,021	-,059	,025	-,047	**-,264***	-,082	,109	,165	-,271	,109	-,026	-,348	-,091	-,311	-,455	**-,370***
**Greasy skin/acne**	-,081	-,124	,080	,066	-,076	,016	-,037	,067	-,166	-,139	**-,545***	-,415	-,573	-,285	-,285	,063	,041	-,250	-,241	-,156
**Varicose veins**	,102	,070	-,027	-,100	-,009	,021	,007	,232	,250	,118						,177	,121	,174	,215	,018
**Brownish marks on face**	-,057	,067	,030	,114	,128	,083	,057	,084	,005	,162						,003	-,053	-,077	-,022	,330
**Itchy skin**	**-,298***	**-,312****	-,053	-,064	-,181	-,044	-,058	,104	,088	,195	-,356	-,548	-,052	-,103	-,190	-,105	-,045	-,204	-,065	,057
**Changes in nipples**	-,011	-,114	-,176	,172	,109	-,106	,027	-,030	-,119	-,032	-,005	-,125	-,206	,094	,056	-,028	,038	-,198	-,124	-,187
**Stretch marks**	-,122	-,121	-,138	,039	,047	-,313	-,317	-,115	-,204	-,031						-,252	-,140	**-,368***	-,231	,055

**Swollen hands or feet**	-,055	-,060	,015	-,053	,039	-,114	-,037	,071	,080	,056	,147	,046	,317	-,391	-,369	-,299	,020	**-,632****	-,287	-,124

*Women who did not experience any of the symptoms did not answer questions about their limitations. Blank space indicates no answer in that item.

Note. Model adjusted for maternal age and body mass index at the 16th or 34th gestational weeks.

Abbreviations: OPF, Overall physical fitness; CRF, cardiorespiratory fitness; MS, Muscular strength; S-A, Speed-agility; FLEX, Flexibility.

*$p < 0.05$; **$p < 0.01$.
